# Challenges to immunization: the experiences of homeless youth

**DOI:** 10.1186/1471-2458-12-338

**Published:** 2012-07-04

**Authors:** Alexander Doroshenko, Jill Hatchette, Scott A Halperin, Noni E MacDonald, Janice E Graham

**Affiliations:** 1 Department of Pediatrics, Canadian Center for Vaccinology, Dalhousie University, Halifax, Nova Scotia, Canada; 2 IWK Health Centre, Halifax, Nova Scotia, Canada; 3 Departments of Sociology and Social Anthropology, Dalhousie University, Halifax, Nova Scotia, Canada

**Keywords:** Homeless youth, Hard to reach population, Vaccines, Immunization programs, Infectious diseases, Invasive meningococcal disease

## Abstract

**Background:**

Homelessness is a critical social issue, both a product of, and contributing to, poor mental and physical health. Over 150,000 young Canadians live on the streets. Homeless youth experience a high incidence of infectious diseases, many of which are vaccine preventable. Early departure from school and limited access to public health services makes them a particularly vulnerable high-risk group. This study explores challenges to obtaining essential vaccines experienced by homeless youth.

**Methods:**

A qualitative research study to explore knowledge, attitudes, beliefs, and experiences surrounding immunization of hard-to-reach homeless youth was designed. Participants were recruited for focus groups from Phoenix House and Shelter, a non-profit, community-based organization assisting homeless youth in Halifax, Nova Scotia, Canada. An experienced facilitator guided the recorded discussions. Transcripts of audiotapes were analyzed using a constant comparative method until data revealed a set of exemplars and themes that best captured participants’ knowledge, attitudes, beliefs and experiences surrounding immunization and infectious diseases.

**Results:**

Important themes emerged from our analysis. Considerable variability in knowledge about immunization and vaccine preventable diseases was found. The homeless youth in the study had limited awareness of meningitis in contrast to a greater knowledge about sexually transmitted infections and influenza, gained during the H1N1/09 public health campaign. They recognized their poverty as a risk for contracting infectious diseases, along with their inability to always employ known strategies to prevent infectious diseases, due to circumstances. They showed considerable insight into the detrimental effects of poor hygiene, sleeping locations and risk behaviour. Interviewed homeless youth regarded themselves as good compliers of health professional advice and offered valuable suggestions to improve immunization in their population.

**Conclusions:**

To provide effective public health interventions, it is necessary to consider the knowledge, attitudes, beliefs, and experiences of hard to reach, high risk groups. Our study shows that homeless youth are interested and capable in discussing immunization. Active targeting of homeless youth for public health immunization programs is needed. Working collaboratively with non-profit organizations that assist homeless youth provides an opportunity to increase their knowledge of infectious risks and to improve immunization strategies in this vulnerable group.

## Background

Homelessness is a critical social issue. While viewed in the past as a plight primarily of men “down on their luck”, women, children and youth now represent its fastest growing subgroups [1-3]. Being homeless has hidden hazards that the expression “living on the streets” overlooks - homeless individuals do not have a secure place to live. They sleep in unsafe, unhygienic buildings and abandoned cars or in shelters where violence and abuse is common and not easily escapable [4]. They experience violence, prostitution, addictions and poor health [5]. Homeless youth are shown to be more vulnerable to nutritional inadequacies compared to the general population [6], use tobacco, alcohol and street drugs at rates substantially higher than non-homeless youth [7] and exhibit higher prevalence of sexual risks, including having multiple sex partners, inconsistent condom use, history of sexually transmitted diseases and “survival sex” or receiving money, food, clothing and shelter in exchange for sex [8,9]. Homelessness in pregnant women leads to the increased risk of unfavorable perinatal events including low birth weight and prematurity in infants [10]. In Canada, 150,000 youth are living on the streets everyday [11,12].

Homelessness increases a person’s exposure to communicable diseases [13]. Rates of several infectious diseases are much higher among homeless and street youth than among their non-homeless peers. North American studies report prevalence of hepatitis B ranging from 3.4% to 13.2% in street youth compared to 0.78% in the same age cohort of the general population. HIV prevalence ranged from 0.5% to 5.8% in street youth compared to 0.2-0.29% in sentinel adolescent clinics and young offenders. Prevalence of hepatitis C ranged from 6.2% to 16.9% in street youth in comparison to 0.1% to 1.51% range in general population [14]. Prevalence of TB infection among homeless youth aged 12 to 25 years in Sydney, Australia is higher than in the general population [15].

Incidence of invasive meningococcal disease (IMD) is shown to be higher in settings of congregate living. Historically, IMD was common among military recruits [16]. The incidence of IMD is higher among college students residing on campus compared to off-campus residents (3.24 per 100,000 vs 0.96 per 100,000) [17]. Outbreaks of IMD were described in association with jails [18]. Homeless youth living in shelters or on the streets share the same characteristics of congregate living that position them at increased risk of IMD and other vaccine-preventable infectious diseases [19]. In Canada, the incidence of invasive meningococcal disease (IMD) in 15–19 year olds is 2.0 per 100,000, second only to infants younger than 1 year old [20] even though IMD caused by serogroups A, C, Y and W135 is preventable by immunization.

Making necessary vaccines both available and accessible to highly vulnerable homeless youth is a critical public health issue. Ensuring equal access to health promotion and disease prevention services for all people is a fundamental tenet of public health. One of the most successful public health interventions, immunization has greatly reduced the incidence of communicable diseases [21]. Together with addressing adverse social circumstances that lead to homelessness, immunization is an effective disease prevention strategy that would improve the health of homeless youth.

In Canada, while a number of publicly funded vaccines are offered to the general population, the largest proportion is administered through childhood immunization programs. The National Advisory Committee on Immunization (NACI) reviews the evidence for effectiveness and safety of vaccines and sets a recommended schedule. However each province defines its own schedule of publicly funded vaccines, based on competing priorities, resulting in different vaccine schedules across the country [22]. Immunization of youth is often carried out through school-based programs that can be delivered systematically by public health services. Alternatively, vaccines can be administered by medical or nursing practitioners in a clinic or office setting often during visits for an unrelated health matter.

Obtaining accurate and complete information on immunization coverage of youth and adults in many Canadian provinces is challenging. A key goal of the Canadian National Immunization Strategy (NIS) is to ensure equitable access to the recommended vaccines across jurisdictions and in special populations [23]. In most provinces and territories, immunization information is collected primarily on children, with variability between jurisdictions with respect to the type of data being collected. Many jurisdictions do not have effective vaccine registries [23]. Challenges are even greater among high-risk homeless youth [24]. Education of homeless youth is often interrupted by suspensions and school drop-outs [25]. The Canadian survey showed that 40.1% of street youth reported that they had dropped out of school permanently and 37% reported that they had been permanently expelled [11]. Homeless youth have also limited access to health care services [26]. Poor access to health care has also been associated with general distrust of adult authority figures, perceived discrimination by health care workers, worries of confidentiality breeches and fears of being reported to the law enforcement authorities [27-29]. Organizational barriers to access health care services also exist for street youth. Living without a fixed address and not having identity papers prevent them from obtaining services in some hospitals and clinics in Canada [28]. Poor access to health care generally translates into missed opportunities for vaccination. Specific to immunization, health care providers’ lack of consistency in vaccine eligibility knowledge [30] and jurisdictional variations in vaccine schedules [31] contribute to fragmented immunization and uncertain vaccine coverage among homeless youth. Health care services at homeless shelters predominately target the management of sexually transmitted infections (STI) and drug treatments, while immunization receives low priority [19,32].

There is a paucity of descriptive empirical studies concerning access of hard to reach groups to vaccines and immunization programs. This study examined the challenges for homeless youth in obtaining essential vaccines. In order to better understand barriers to their accessing recommended vaccines, homeless youth were asked to share their knowledge, attitudes and beliefs surrounding infectious diseases, and experiences in accessing immunization. The research team was particularly interested in identifying homeless youth’s understandings of lesser known vaccine preventable diseases, such as IMD which has the second highest peak in disease incidence in the age range of homeless youth and for which they are at increased risk due to their congregate living. Moreover vaccination against IMD is offered in adolescence, the age of homeless youth. This study was designed to bridge the gap in existing knowledge on immunization of homeless youth. This study was approved by the IWK Health Centre Research Ethics Board in Halifax, Nova Scotia, Canada.

## Methods

### Study design and rationale

This was a qualitative exploratory study conducted among homeless youth using focus group discussions to explore their knowledge, attitudes, beliefs and experiences surrounding infectious diseases, vaccination and health care. Broad, commonly understood definitions of knowledge as familiarity, awareness and understanding gained through experience and study; attitude as a way a person views something or tend to behave towards it; belief as a principle, proposition or opinion accepted as true; and experience as direct personal participation and observation were used [33]. Prior experience has shown that focus groups work well for involving hard-to-reach community members in program planning and evaluation [34].

### Participants

Participants were recruited from Phoenix House and Phoenix Youth Shelter in Halifax, Nova Scotia, Canada. Phoenix is a non-profit, community-based organization assisting homeless youth through an extensive range of programs and services. Phoenix House is a 10-bed residential facility offering safe, supportive and long-term housing to five young men and women. The average age of residents is 17.8 years and the average length of stay is eight months [35]. Phoenix’s Youth Shelter is a 20-bed facility providing 15 male and five female homeless youth with a short-term emergency housing and is full almost every night of the year [36].

Individuals aged 15 to 24 years who used the Phoenix House or Phoenix Youth Shelter program were recruited. Those in a mainstream educational program, with full-time employment, exhibiting intoxication or having behavioural difficulties were excluded.

### Study process

Posters describing the study were placed in Phoenix House and staff brought the study to the attention of youth through word of mouth. Recruitment was based on convenience sample. Modest incentives in the form of pizza, soft drinks and coffee shop vouchers were offered to participants. Once a sufficient number of youth signed up, a focus group was scheduled. Focus groups continued until the responses achieved saturation, when no new information was being obtained from the sessions. A total of 29 people agreed to participate, 13 of whom were females. Four focus groups of 5 to 10 participants each were conducted during July and August 2009, the period between the first and second waves of the H1N1/09 pandemic. Approximately one quarter of the participants were physically living on the streets, while the others had sought temporary shelter. Two participants identified themselves as having a history of incarceration, and one travelling couple (male and female) had just arrived in Nova Scotia. All participants provided written consent prior to the focus group. Study participants who preferred not to disclose their names were offered to place a mark on the consent form. There were no illiterate participants.

The sessions, lasting from ½ to 2 hours, were conducted on weekdays at the Phoenix House and Shelter, and were digitally recorded and transcribed. Focus groups were led by an experienced facilitator skilled in communicating with at-risk groups. Attention was verbally redirected from more dominant talkers to others in the group, and shy participants were drawn out with questions and encouragement to elaborate. Researchers, including an infectious diseases physician, were present during the focus groups and all were available afterwards to answer questions and continue discussions on a one-to-one basis.

Following the guidelines of Krueger & Casey [37], conversations began with general questions about infectious diseases, prevention strategies, immunization and the health care system, with a gradual transition to more specific questions about lesser known diseases such as meningitis. Questions in subsequent groups were modified after reflection of the information and themes emerging in the previous group discussions.

### Analysis

Transcripts of audiotapes of each focus group session and notes prepared by the researchers were analyzed using a constant comparative methodology that identifies and compares concepts, experiences and activities that arose in the conversations, assessing both common and divergent emerging themes [38]. Review of the transcripts was undertaken independently by two investigators. The textual data were assessed in the form of partial and/or full sentences that indicated similarities among participants across knowledge, attitudes, beliefs and experiences related to general and specific infectious diseases and immunization. Similar knowledge, attitudes, beliefs and experiences were then organized under emerging themes. Themes and categories were restructured until the textual data revealed a set of exemplars that best captured the knowledge, attitudes, beliefs, and experiences of the participants surrounding infectious disease prevention.

## Results

A comfortable, semi-structured discussion was facilitated among the focus group participants, with probing to elicit the following areas of research interest: knowledge about infectious diseases, vaccines and healthcare, attitudes towards immunization and healthcare system, beliefs that pose potential obstacles to immunization, experiences conducive to contracting infectious diseases and precluding immunization, as well as suggestions to improve immunization. Common themes emerged and are discussed below:

1. Knowledge of infectious diseases, vaccines and healthcare

(a) Identifying infectious diseasesMost of the 29 participants had a good general knowledge about infectious diseases, recognizing that they are contracted from other people. They had a more vague understanding of modes of transmission (i.e. coughs and sneezes, by contaminated hands or objects, and sexually). They were highly aware of STI and of conditions that were receiving wide coverage in the press (e.g. H1N1 pandemic influenza received significant publicity during the late summer of 2009). Although nobody spontaneously identified meningitis as an infectious disease, as the conversation progressed and examples of less common infectious diseases were introduced into the discussion, several individuals proved to have had personal experience and some basic knowledge of meningitis.Those most aware of meningitis had either contracted it personally, had been diagnosed with suspected meningitis or received medication as a result of exposure.Lack of knowledge about meningitis was common.

(b) Perceived risk factors for infectious diseasesRespondents recognized a number of infectious disease risks they encounter daily. They discussed the details and consequences of living in poverty, such as having little access to clean washing facilities, eating irregularly and sharing close personal space with other people.They recognized sub-standard accommodations as an infectious disease risk factor, providing accounts of “cardboard apartments”, “boarding houses”, “garbage cans”, “open grassy fields”, “alleyways”, “abandoned cars” and “couch surfing” [temporarily staying with friends or family].They spontaneously cited hepatitis, mononucleosis and influenza as the diseases putting them at most risk. While the conversation began with general comments about their vulnerability to these more common infections, later other infectious diseases were incorporated into the discussion among the participants.

(c) Prevention strategiesParticipants were well aware that infectious diseases can be harmful and expressed concern about prevention.Knowledge of prevention strategies was congruent with perceived risk factors.While possible prevention practices were known, limited ability to employ these strategies was recognized as a barrier. The more urgent need to survive the contingencies of life on the streets took priority and varied with the degree of homelessness.

(d) Knowledge about vaccines and immunizationParticipants acknowledged that vaccines were protective.Our participants lacked information about the different types of vaccines and frequent confusion concerning preventative antibiotics occurred.When asked about which vaccines they had received, the majority of participants mentioned hepatitis B and influenza. Most individuals remembered vaccines received in schools and two participants recounted being immunized in jail. There were frequent misconceptions and confusion between seasonal and “swine” influenza (H1N1 pandemic influenza).Consistent with the lack of general awareness of meningitis disease, noted in section 1 (a), nobody mentioned meningitis vaccine without prompting.

2. Attitudes towards immunization and healthcare

(a) Attitudes towards vaccinesThere was an ambiguous expression of attitudes pertaining to vaccines. The majority of the study participants said they would be willing to be immunized despite having reservations about whether the vaccine actually worked. They were active compliers of health professional advice, responding positively to immunization decisions when such opportunities were made available.While some side effects of vaccination including fever, rash, swelling, nausea, headache and feeling faint or anxious were identified, nobody mentioned anaphylaxis.Many youth mentioned that safety of vaccines depends on individual factors.Only a few explanations were provided about why youth would not have a vaccine. Among them were fear of needles and concerns about its effectiveness.

(b) Attitudes towards healthcareParticipants expressed mixed feelings about the health care system, in common with many Canadians, especially concern about the availability of family doctors. Several said that the health care system “sucked”, citing limited access.There was a concern among those who travelled that provincial health insurance plans do not provide medical coverage in other provinces without bureaucratic hassles. Among some, there was a lack of understanding about how the health system works, e.g., how to get a provincial health insurance card.Balancing the negative comments, several participants made positive statements about the health care system and how they were treated.Several homeless youth valued the added security of the “ability to sleep in the hospital” as a good thing.

(c) Intention to get immunizedParticipants indicated that they would get immunized if it was readily available and easily accessible, but they would not go to lengths to get a shot.

3. Beliefs

(a) Caring for youth is not a priority for the health care systemMany participants self-identified as social pariahs. They believe that providing healthcare for them is not high on the agenda of healthcare professionals and services.Others saw their homelessness as largely invisible, and therefore not presenting any barriers when they sought medical attention at a walk-in clinic or hospital emergency, for example.

(b) Getting immunized is not a priority for homeless youthParticipants mentioned that other aspects of their personal lives take precedence over getting proper health care including vaccination.

4. Experiences associated with infectious diseases and their prevention

(a) Living conditionsParticipants recognized that their living conditions and lack of affordable accommodation placed them at a higher risk for contracting infectious diseases.

(b) Incarceration and immunizationTwo youth acknowledged being immunized while incarcerated.

(c) Irregular school attendanceSome recalled being immunized in schools; others became homeless during their school years or had irregular attendance and were missed by immunization programs. Homeless youth had a history of truancy, precluding them from the usual sites for vaccination programs.

(d) Opportunities to get vaccinatedMany said that they take advantage when vaccines are made available, regarding it as an opportune event.

5. Barriers to immunization

(a) Lack of communication and informationWhile it is widely held that homeless youth rely on information from their “inner circle” rather than from health professionals [39], study participants said that they readily comply with health care providers’ advice. They emphasized that lack of communication from their health providers was the reason they did not get immunized.

(b) CostCost was seen as a major deterrent to immunization to the homeless youth. They felt that immunization should be free irrespective of their ability to pay, and they said they would not be able to afford vaccines if they had to pay.Several youth, particularly females discussing the HPV immunization, mentioned that they would pay a nominal fee (“up to $20”) for a vaccine that they really wanted. There were persistent misunderstandings, however, as to which vaccines were free.

(c) Vaccination policy and lack of access to immunization servicesMany travellers and those without family doctors said that they did not have access to immunization facilities. The only options open to them were walk-in clinics and Phoenix House.Uncertainty among some health care providers concerning vaccine access, demographic targeting and immunization campaigns contributes to public uncertainty. Youth reported being told that they were not eligible for free influenza immunization.

6. Solutions to improve immunization

(a) Better outreach using appropriate mediaOur study participants recommended that better advertisement of time and location of free immunization sites would improve their awareness of what is available to them. They suggested advertising in public and commercial locations, including on buses and beverage containers (commonly mentioned in humor), and pamphlets in grocery stores and added to grocery bags.Participants had extensive awareness and access to e-mail, citing Phoenix House, Public Archives and the local university libraries for example, and many use social networking media such as “Facebook” or “My Space”.Media savvy, they insightfully suggested that advertisements should be conducted in a positive rather than threatening fashion.

(b) Health care system should be more homeless youth friendlyMany youth felt that health professionals should do more to accommodate the needs of the homeless and wanted to see more health care options open to them.

(c) Improved accessParticipants suggested shelters such as Phoenix House as an access point for vaccines.

## Discussion

Although a number of studies address strategies to meet the health care needs, including delivery of vaccinations, of young people living in high-risk settings such as homeless shelters, residential facilities and prisons [19,40,41], hard-to-reach youth are rarely included in developing these strategies. No studies, to our knowledge, specifically explore the knowledge, attitudes, beliefs and experiences of homeless youth about lesser known vaccines, such as those for invasive meningococcal disease. Our qualitative research provides candid insights from the youth themselves, into challenges they face with regards to infectious diseases, in general, and vaccination against lesser known diseases such as meningococcal disease, in particular. While the pandemic could not have been anticipated in the early design of the study, it provided an unexpected opportunity for comparing awareness of various infectious diseases. Importantly, immunization is a low priority for homeless youth; finding food, shelter and employment is their pressing preoccupation. Their lack of awareness about certain infectious diseases, however, also contributes to low vaccine uptake. During the focus group discussions, STIs, hepatitis B and mononucleosis were acknowledged spontaneously. Meningitis received no mention without prompting by the researchers, and knowledge of this preventative vaccine was limited. The study took place between the first and second waves of the 2009 H1N1 influenza pandemic. While there was wide awareness of influenza, there was some confusion about the differences between seasonal and H1N1/09 pandemic influenza. Clearly, public information campaigns are effective in educating the hard to reach. Targeting the sites that are homeless youth-friendly, and using popular media to convey information about where immunizations can be obtained, would improve awareness of meningitis and other vaccine preventable vaccines in this vulnerable group.

The homeless youth held the perception that health professionals were not interested in offering vaccines to them. Indeed, there is a lack of clarity around national and provincial recommendations regarding meningococcal immunization of homeless. In Canada it is recommended that all adolescents and young adults should be immunized with meningococcal C conjugate vaccine [42]. Homeless youth, however, are not specifically named as a high-risk group. As a result, health professionals do not often follow-up on meningitis immunization among the youth they come in contact. Additionally, the assumption that homeless youth have already been immunized while in school is erroneous. Currently in Nova Scotia it is recommended that adolescents in grade 7 be immunized against meningitis [43]. Consideration should be given to extend this recommendation to homeless youth beyond 16 years as school-based immunization programs will have missed many.

Alternative facilities to deliver immunization to homeless youth should be considered. Most participants in this study felt that the best place for them to get information and be immunized was Phoenix House, which offers an extensive network of services to homeless youth, is well known and trusted, and has gained considerable street ‘cred’(ibility). Public health providers might explore forging a working relationship with non-profit organizations caring for homeless youth who often have on-site nurses who could readily provide vaccine coverage to this vulnerable population. Vaccine education should also be made available to youth while in jail. The incarcerated youth interviewed had received vaccines in jail but were uncertain what they were. Unfortunate social scenarios may provide a public health opportunity; administration of vaccines in jail should be documented, reported to Public Health authorities and time taken to ensure the youth understand what they have received.

Regarding cost, a distinction between free vaccines and vaccines that are currently not covered by the public health plans should be clearly explained to youth by health professionals. There is a variation in eligibility for publicly funded vaccines between jurisdictions and over time. Lack of clarity on this matter can lead to the inaccurate impression that youth have to pay for vaccines, a major deterrent to vaccination in this population. This could be the case with publicly funded vaccine for all adolescents and young adults with meningococcal C conjugate vaccine, and quadrivalent meningococcal conjugate vaccine administered to certain high risk groups.

Our study has several limitations. Individuals who were recruited to participate in this research exhibited a diverse range of knowledge, attitudes, beliefs and experiences. The study was conducted between the first and second waves of the H1N1/09 pandemic when the influenza public health campaign was fresh and seen to have been quite effective in reaching homeless youth. Focus groups were conducted during the summer months and the participants may not have been representative of participants during other seasons, as the street population is very fluid. Only a quarter described themselves as physically living on the streets; the remaining viewed themselves as only in temporary need of shelter. Certain characteristics of homeless youth like duration of their homelessness, education level or health status were not actively and quantitatively explored. While collective opinion of homeless youth was sought, future research might address how individual characteristics influence their knowledge, attitudes and beliefs related to immunization. Some opinions expressed during focus groups were based on inaccurate information that posed challenges for interpretation.

## Conclusions

Delivering vaccines to hard-to-reach populations has always been a challenge. In Canada, some publicly funded vaccines are scheduled in school cohorts and family practices not used by homeless youth. Challenges to identifying and locating hard to reach youth, lack of awareness about immunization sites and essential vaccines on the part of youth, and passive approaches taken by health professionals contribute to this population being missed. A clear finding from this study is that active targeting of homeless youth for immunization against infectious diseases is needed and would be largely welcomed. Knowledge varied widely among homeless youth about infectious diseases, with a palpable lack of knowledge about meningitis and better awareness about STIs and influenza. All study participants recognize, however, that they live in insecure locations and they stated emphatically they are ready candidates for health care advice and easily accessible interventions such as immunization. In an era when there is increased recognition of the value of patient-centered care, the particular knowledge, attitudes, beliefs and experiences of homeless youth should be taken into consideration when developing vaccination policies both at the national and provincial levels for this hard to reach group. Policymakers should consider immunization challenges faced by homeless youth in formulating policies and recommending programs. Working collaboratively with non-profit organizations assisting homeless youth provides a valuable and rich opportunity to increase knowledge of infectious risks and to improve immunization in this vulnerable group. Homeless youth know well the most effective way to reach them. Public health policymakers should be encouraged to listen to the voices of the most vulnerable and attend to their suggestions, and to design and build programs that better understand and meet their needs for healthy living and social inclusion.

## Competing interests

AD currently works for a provincial public health authority. The authors declare that they have no other competing interests.

## Authors’ contributions

SAH and NMD conceived the idea for this study and were the fellowship supervisors of AD. AD conceptualized and designed the study, participated in the focus groups, analyzed findings and wrote the initial draft of the article. JH and JG refined the study methodology and analysis plan and also participated in the focus groups. All authors provided input on study methodology and analysis and helped review, revise and edit the article. All authors read and approved the final manuscript.

## Authors’ information

At the time of this study AD was with the Canadian Center for Vaccinology, Dalhousie University and the IWK Health Centre, Halifax, NS, Canada. SAH and NMD are with the Canadian Center for Vaccinology, Dalhousie University and the IWK Health Centre, Halifax. JH is with the IWK Health Centre, Halifax. JG is the Canada Research Chair in Bioethics with the Canadian Center for Vaccinology and Professor in Departments of Pediatrics (Infectious Diseases) and Sociology and Social Anthropology, Dalhousie University and the IWK Health Centre, Halifax.

## Pre-publication history

The pre-publication history for this paper can be accessed here:

http://www.biomedcentral.com/1471-2458/12/338/prepub
